# MicroRNA-532-5p Regulates Pericyte Function by Targeting the Transcription Regulator BACH1 and Angiopoietin-1

**DOI:** 10.1016/j.ymthe.2018.08.020

**Published:** 2018-09-01

**Authors:** Sadie C. Slater, Eva Jover, Andrea Martello, Tijana Mitić, Iker Rodriguez-Arabaolaza, Rosa Vono, Valeria V. Alvino, Simon C. Satchell, Gaia Spinetti, Andrea Caporali, Paolo Madeddu

**Affiliations:** 1 Bristol Heart Institute, Translational Health Sciences, University of Bristol, Bristol Royal Infirmary, Bristol BS2 8HW, UK; 2 University/BHF Centre for Cardiovascular Science, Queen’s Medical Research Institute, University of Edinburgh, Edinburgh EH16 4TJ, UK; 3 Laboratory of Cardiovascular Research, IRCCS MultiMedica, Milan 20138, Italy; 4 Bristol Renal, Translational Health Sciences, University of Bristol, Whitson Street, Bristol BS1 3NY, UK

**Keywords:** angiogenesis, pericytes, microRNA

## Abstract

MicroRNAs regulate endothelial function and angiogenesis, but their implication in pericyte biology remains undetermined. A PCR array, covering a panel of 379 human microRNAs, showed microRNA-532-5p to be one of the most differentially modulated by hypoxia, which was confirmed by qPCR in both skeletal muscle and adventitial pericytes. Furthermore, microRNA-532-5p was upregulated in murine muscular pericytes early after experimentally induced ischemia, decreasing below baseline after reperfusion. Transfection of human pericytes with anti-microRNA, microRNA-mimic, or controls indicates microRNA-532-5p modulates pro-angiogenic activity via transcriptional regulation of angiopoietin-1. Tie-2 blockade abrogated the ability of microRNA-532-5p-overexpressing pericytes to promote endothelial network formation *in vitro*. However, angiopoietin-1 is not a direct target of microRNA-532-5p. *In silico* analysis of microRNA-532-5p inhibitory targets associated with angiopoietin-1 transcription indicated three potential candidates, *BACH1*, *HIF1AN*, and *EGLN1*. Binding of microRNA-532-5p to the *BACH1* 3′ UTR was confirmed by luciferase assay. MicroRNA-532-5p silencing increased BACH1, while a microRNA-532-5p mimic decreased expression. Silencing of *BACH1* modulated angiopoietin-1 gene and protein expression. ChIP confirmed BACH1 transcriptional regulation of angiopoietin-1 promoter. Finally, microRNA-532-5p overexpression increased pericyte coverage in an *in vivo* Matrigel assay, suggesting its role in vascular maturation. This study provides a new mechanistic understanding of the transcriptional program orchestrating angiopoietin-1/Tie-2 signaling in human pericytes.

## Graphical Abstract

**Figure:**
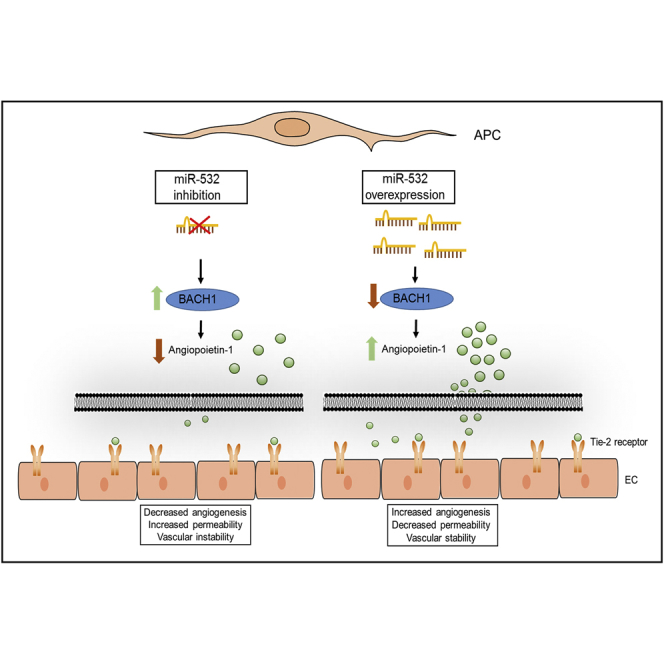

## Teaser

Angiopoietin-1 is an important regulator of angiogenesis, yet little is known regarding its transcriptional regulation. Slater et al. (2018) demonstrate angiopoietin-1 induction in pericytes occurs via miR-532-5p regulation of BACH1 and modulation of this pathway promotes changes in endothelial permeability, vascular stability, and angiogenesis.

## Introduction

Angiogenesis is a tightly regulated process that involves the interaction between endothelial cells (ECs) and pericytes (PCs).^1^ Current mechanistic understanding indicates the canonical angiopoietin (Ang)/Tie-2 pathway plays a key role in the paracrine cross-talk between the two cell populations.^2^, ^3^, ^4^ Tie-2 expressed by ECs is activated by PC-secreted Ang-1, resulting in the induction of downstream pathways mediating survival, proliferation, migration, and anti-inflammatory signals. In contrast, Ang-2 acts as a partial agonist of Tie-2, inhibiting Tie-2 signaling in the presence of Ang-1, but activating Tie-2 in the absence of Ang-1.^5^ Surprisingly, little is known about the transcriptional regulation of the Ang/Tie-2 pathway.

MicroRNAs (miRs) are small noncoding RNAs that function in diverse biological processes via post-transcriptional gene regulation. They modulate protein expression by binding to complementary or partially complementary target sites in the 3′ UTRs of mRNA, causing inhibition of gene expression.^6^ miRs have been identified in cells,^6^ serum, and plasma.^7^ Some miRs remain within the cell-modifying transcriptional regulation, while others are released, acting like paracrine or endocrine factors.^8^, ^9^ Changes in the expression profile of several miRs have been associated with cardiovascular processes and diseases.^10^, ^11^ In particular, a class of miRs is specifically modulated by hypoxia and implicated in the regulation of angiogenesis. These hypoxia-miRs are in the focus of intense research and represent potential targets for the treatment of ischemic disease.^12^ Nevertheless, a few studies have addressed the participation of miRs in the regulation of PC functions.^13^, ^14^, ^15^, ^16^ Moreover, although some data exist on the hypoxia-induced Let-7d having a role in PC differentiation,^17^ no systematic analysis has been carried out to characterize the profile of hypoxia-miRs that target angiogenic genes in human PCs.

The present study investigates the effect of hypoxia on global miR expression in a subset of human CD34^+^ and CD146^−^ PCs isolated from the saphenous vein—adventitial PCs (APCs). Within the miRs identified to be differentially modulated by hypoxia, we focused on miR-532-5p because, while being reportedly expressed in cancer,^18^ rheumatoid arthritis,^19^ epilepsy,^20^ and deep vein thrombosis,^21^ its cardiovascular role remains undetermined. Interestingly, a recent study showed miR-532-5p exerts a protective action in a model of myocardial infarction by repressing maladaptive transition of coronary ECs to a fibroblast-like phenotype via endothelial-to-mesenchymal transition.^22^ Results here show for the first time a new molecular mechanism centered on miR-532-5p, which by inhibiting the transcriptional repressor BTB and CNC homology 1 (BACH1) induces the expression and secretion of Ang-1 by human PCs. Activation of this signaling mechanism results in autocrine inhibition of PC apoptosis and paracrine activation of EC migration and angiogenic activity.

## Results

### Characterization of Cell Phenotype

PCs are classically identified as mural cells surrounding capillaries and arterioles in tissues and vasa vasorum in the adventitia of large vessels. Both populations express PDGFR-β, CSPG4 (NG2), ACTA2 (SMA), Desmin, T-box family transcription factor (Tbx18), and CD248 but differ with regard to CD146, which is acknowledged as a distinctive marker for muscle PCs (MCPs).^2^, ^23^, ^24^ Here, we confirmed the typical phenotype of expanded human PCs by flow cytometry and immunocytochemistry. Saphenous vein-derived APCs express PDGFR-β, CD90, CD73, CD105, and CD44 but are negative for CD34, CD31 (PECAM-1), CD146, and CD45 (Figure S1A). Furthermore, APCs stain positive for PDGFR-β, GATA-4, and vimentin, but do not express VE-cadherin (Figure S1B). The typical profile of PCs from human skeletal muscles (MPCs) was verified by immunocytochemistry for NG2, PDGFR-β, CD146, and CD56 and flow cytometry identification of alkaline phosphatase (ALP). Additionally, MPCs were negative for the endothelial antigen CD31 and the satellite cell marker PAX7 (Figure S1C).

### Effect of Hypoxia on miR Profile in Human APCs

We first used a PCR array, covering a panel of 379 most common human miRs, to assess the change in the APC miR profile from normoxia to hypoxia (48 hr, 2% O_2_). Controls (“no template” sample in the reverse transcription step [negative control] and RNA spike-in [technical control]) indicated good performance of the profiling experiment. Four APC cell lines, each derived from individual patient saphenous vein samples, were tested. The array detected 175 miRs, in all studied cell lines, with an average of 209 being detected per sample (data not shown). When comparing normoxic to hypoxic samples using a paired t test, 19 miRs were found to be differentially expressed using a cutoff p value < 0.05 (Table 1). Due to the small number of patient samples used for the array, no multiple comparison correction was applied. Of the 19 miRs, 4 were upregulated and 15 downregulated in response to the hypoxic stimulus. The first four top hits in the list (miR-210, miR-98-5p, miR-192-5p, and let-7f-5p) have been previously associated with angiogenesis.^25^, ^26^, ^27^, ^28^ Interestingly, however, we could not find any published data about an association of miR-532-5p, the fifth top hit, with human PCs, hypoxia, or angiogenesis.

**Table 1:** Individual Results for the Top 19 Most Differentially Expressed miRs

miR Name	SD	Fold Change (2^−Δ*ct*^)	p Value
hsa-miR-210	1.5	−3.2	0.0072
hsa-miR-98-5p	0.14	1.3	0.010
hsa-miR-192-5p	0.51	−2.5	0.014
hsa-let-7f-5p	0.13	−1.2	0.017
hsa-miR-532-5p	0.16	1.3	0.021
hsa-miR-99b-5p	0.12	1.2	0.024
hsa-let-7d-5p	0.13	1.2	0.027
hsa-miR-18a-5p	0.090	1.1	0.028
hsa-miR-432-5p	0.11	1.2	0.030
hsa-miR-539-5p	0.23	1.4	0.030
hsa-miR-16-5p	0.066	1.1	0.030
hsa-miR-299-3p	0.55	−2.1	0.030
hsa-miR-149-5p	0.35	1.6	0.031
hsa-miR-196b-5p	0.085	1.1	0.031
hsa-miR-128	0.19	1.3	0.033
hsa-miR-654-5p	0.40	1.7	0.033
hsa-miR-339-5p	0.27	1.4	0.042
hsa-miR-423-5p	0.26	1.3	0.047
hsa-miR-30c-2-3p	0.44	2.3	0.049

**Figure 1:**
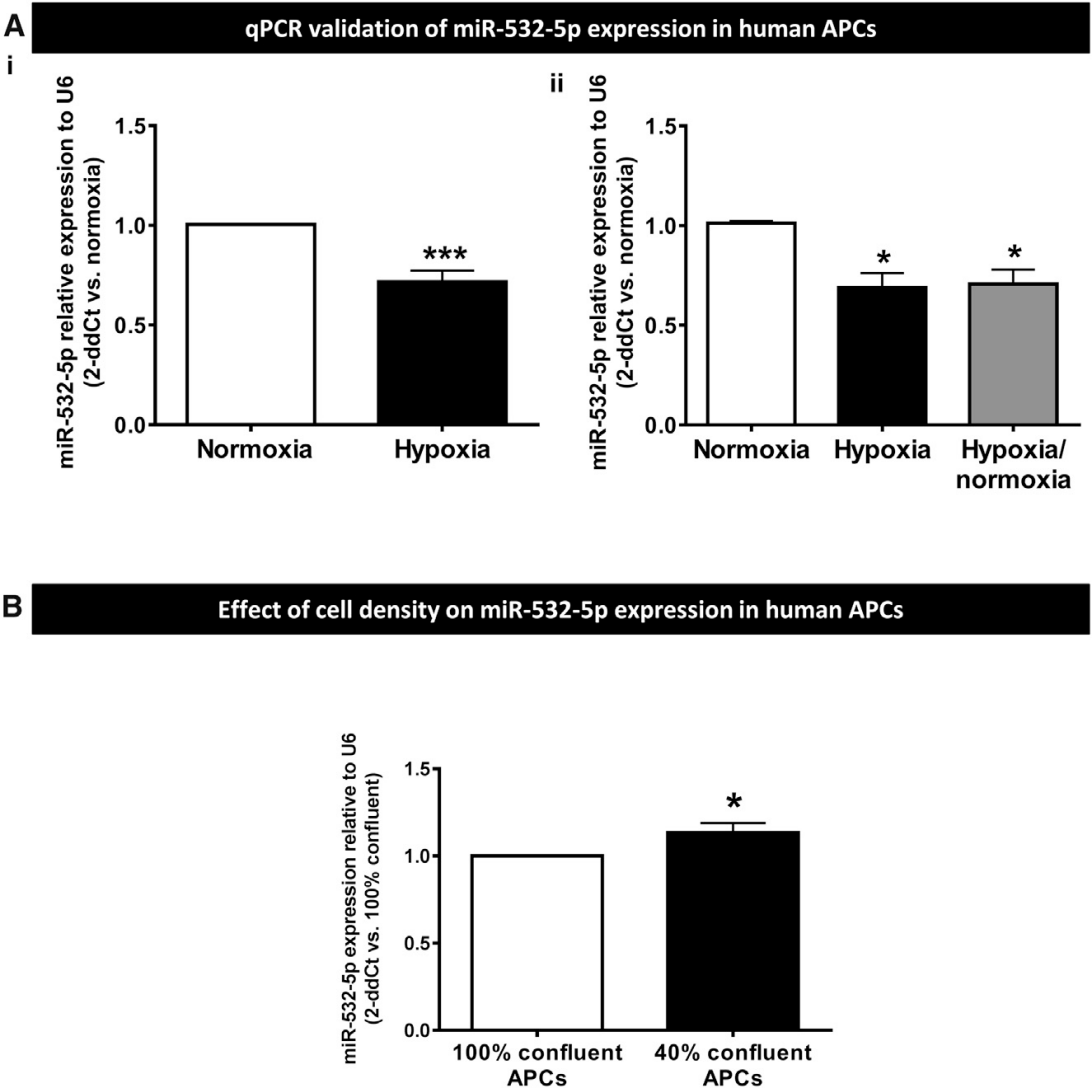
Effect of Hypoxia on miR Profile in Human APCs (A) Validation of miR532-5p expression by qPCR confirms reduction of intracellular levels following APCs’ exposure to 48 hr hypoxia (i, n = 6 biological replicates) or 48 hr hypoxia followed by return to 48 hr normoxia (ii, n = 4 biological replicates). *p < 0.05 and ***p < 0.001 versus normoxia. (B) MiR-532-5p expression is also affected by cell density (n = 4 biological replicates). *p < 0.05 versus confluent APCs. Values are means ± SE.

Validation of the miR array by qPCR confirmed miR-532-5p downregulation in APCs (passage 7) exposed to hypoxia for 48 hr (−29%, p < 0.001 versus normoxia; Figure 1A), this effect persisting after return of cells from hypoxia to normoxia for 48 hr (p < 0.05 versus basal normoxia or hypoxia; Figure 1B). We also found the depressant effect of hypoxia is reproduced at early stage of APC expansion (passage 3; data not shown).

Changes in the degree of mutual cell contacts, such as following PC detachment from neighboring ECs at the initial stage of angiogenesis or *in vitro* culture at different cell densities, reportedly affect the gene expression and expansion capacity.^29^, ^30^ Consistently, we found that cell density significantly affects the miR-532-5p expression of human APCs (Figure 1B). Overall, these data indicate intracellular miR-532-5p levels are differentially modulated by changes in the culture microenvironment.

### Effect of Hypoxia and Ischemia on miR-532-5p in Human MPCs

**Figure 2:**
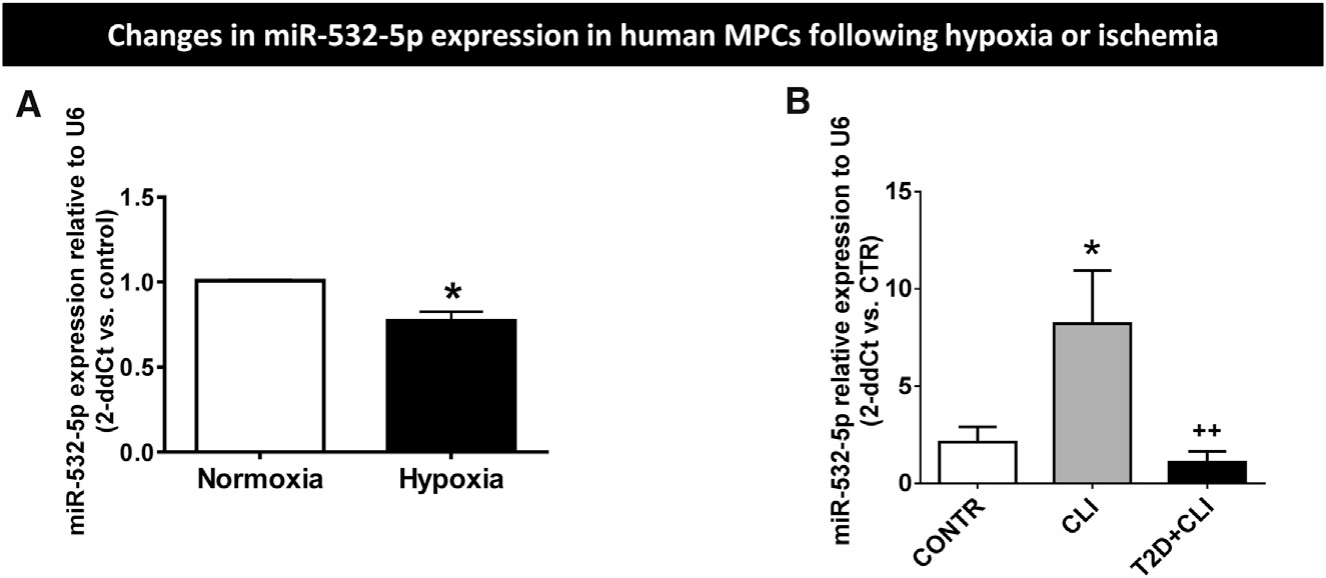
miR-532-5p Levels in Human MPCs (A) Effect of 48 hr normoxia or hypoxia (2% O_2_) on miR-532-5p levels in human MPCs (n = 4 biological replicates). (B) Levels of miR-532-5p in MPCs harvested from healthy (CONTR) or ischemic muscles (CLI) with or without superimposed diabetes (T2D) (n = 5, 3, and 5, respectively). *p < 0.05 versus CONTR, ^++^p < 0.01 versus CLI. Values are means ± SE.

We next investigated if hypoxia modulates miR-532-5p in PCs derived from another anatomical source, skeletal muscles. Results confirm hypoxia reduces the levels of miR-532-5p in human MPCs (Figure 2A). Next, we asked if miR-532-5p expressional changes following *in vitro* hypoxia are reproduced in MPCs freshly harvested from ischemic muscles. To this aim, we compared the miR levels in MPCs isolated from muscular biopsies of healthy subjects (CONTR) or critical limb ischemia (CLI) patients with or without concomitant type 2 diabetes mellitus (T2D) a condition known to jeopardize vascular repair and PC functions (Figure 2B).^31^, ^32^, ^33^, ^34^ Intriguingly, we found that miR-532-5p levels are markedly higher in MPCs from CLI muscles (4-fold, p < 0.01 versus CONTR), with the effect being abolished by coexisting T2D (p = N.S. versus CONTR; p < 0.05 versus CLI).

**Figure 3:**
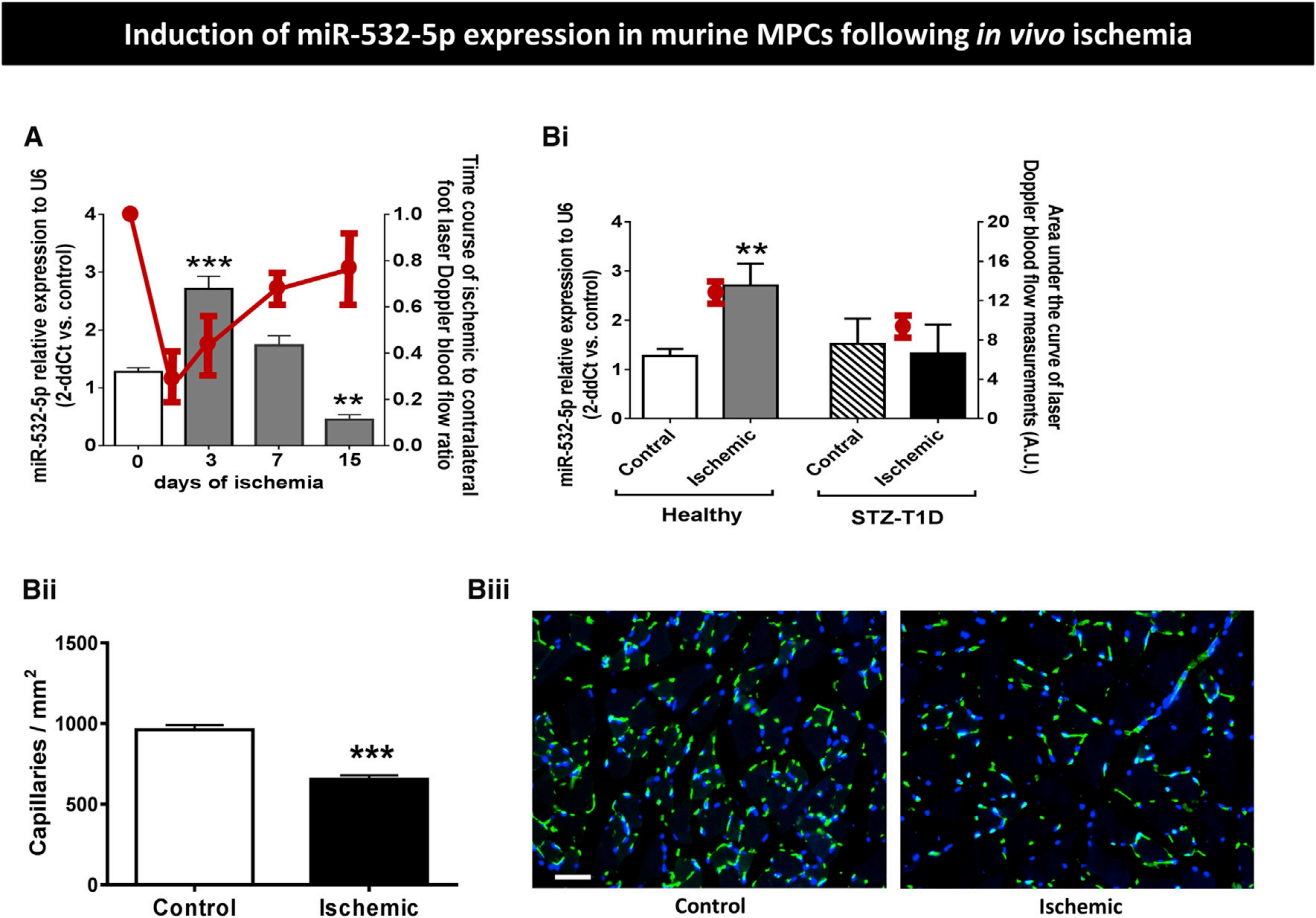
miR-532-5p Levels in Murine MPCs (A) Bar graph showing the time course of miR-532-5p expression in murine NG2^+^ MPCs freshly sorted from muscles of mice subjected to unilateral limb ischemia. The red line graph illustrates the corresponding time course of laser Doppler blood flow measurements, which shows a trend opposite to that of miR-532-5p (n = 4 biological replicates, **p < 0.01 and ***p < 0.001 versus time 0). (B) (i) Bar graph showing the miR-532-5p expression levels in NG2^+^ MCPs freshly sorted from ischemic and contralateral muscles of healthy or streptozotocin-induced type 1 diabetic mice (STZ-T1D) at 3 days from induction of limb ischemia. The red circles represent the area under the curve of laser Doppler blood flow measurements expressed as arbitrary units (AU; n = 4 biological replicates, **p < 0.01 versus MPCs isolated from contralateral non-ischemic muscle). (ii) Bar graph showing capillary density in contralateral muscle 15 days post-ischemia (n = 4, ***p < 0.001 versus control. Values are means ± SE. (iii) Representative images of immunohistological analysis of capillary density. Green staining, isolectin B4; blue staining, DAPI. Scale bar, 50 μm.

We verified the upregulation of miR-532-5p by ischemia by assessing the miR levels in immunosorted NG2^+^ MPCs from our recent study in a murine model of unilateral limb ischemia.^15^ Results confirmed the remarkable upregulation of miR-532-5p in MPCs from ischemic muscles of otherwise healthy mice (Figure 3A). The levels of miR-532-5p peaked at day 3 post-ischemia (2.5-fold, p < 0.0001 versus time 0) and then decreased to below the baseline values by day 15 post-ischemia (p < 0.01 versus time 0). Interestingly, the time-based changes in miR-532-5p were contrary to the Doppler blood flow changes (expressed as ischemic to contralateral blood flow ratio), thus indicating an inverse relationship between the two measurements (Figure 3A). Furthermore, we compared the levels of miR-532-5p in NG2^+^ MPCs sorted from ischemic and contralateral limb muscles of healthy and streptozotocin (STZ)-induced type 1 diabetic (T1D) mice. Results indicate the inductive effect of ischemia on miR-532-5p was completely abrogated by the concomitance of diabetes (Figure 3Bi). This inhibitory effect of T1D on miR-532-5p was paralleled by a worse perfusion recovery, as assessed by calculating the area under the curve of laser Doppler blood flow measurements over 15 days post-ischemia (Figure 3Bi) and by a reduced microvascular capillary density (Figures 3Bii and 3Biii), as assessed by immunohistochemistry (653 ± 25 versus 960 ± 30 capillaries/mm^2^ in healthy mice, p < 0.001).

### Autocrine and Paracrine Effects of miR-532-5p Modulation in Human APCs

**Figure 4:**
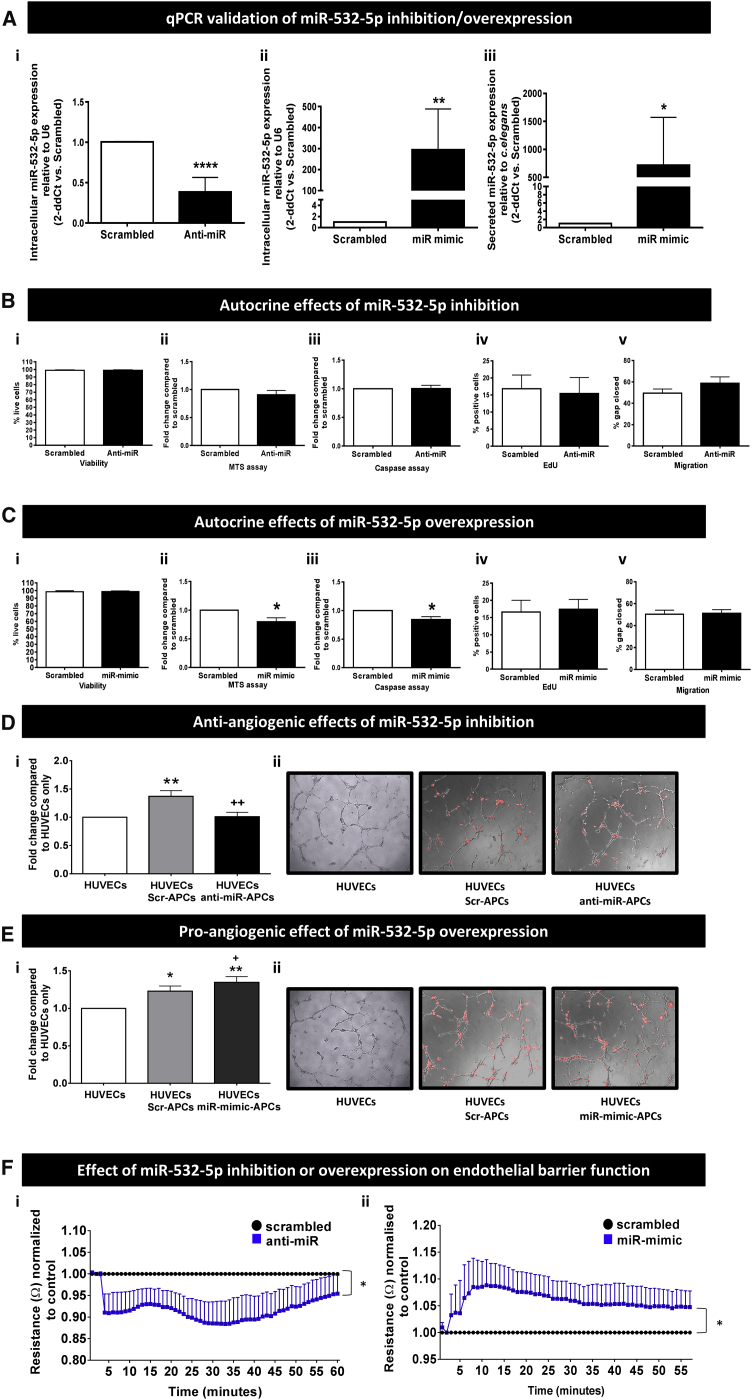
Effect of Forced Modulation of miR-532-5p Expression (A) Confirmation of effective transfection by miR modulators, with anti-miR causing miR inhibition (i) and miR mimic causing increased miR expression intracellularly and in conditioned medium (ii and iii); n = 8 biological replicates. *p < 0.05, **p < 0.01, ****p < 0.0001 versus scrambled. (B) Inhibition of miR-532-5p does not affect APC functions. (C) Forced expression of miR-532-5p induces a decrease in metabolic activity and apoptosis; n = 6 biological replicates, *p < 0.05 versus scrambled. (D and E) The effect of miR-532-5p modulation on network formation was investigated in a Matrigel assay. (D) (i) Co-culture of miR-532-5p-inhibited APCs with HUVECs significantly decreased the ability of the latter to form networks on Matrigel compared to scrambled-treated APCs; n = 9 biological replicates, **p < 0.01 versus HUVECs alone, ^++^p < 0.01 versus scrambled-APCs. (ii) DiL (red) staining of APCs shows miR-532-5p inhibition resulted in no change in the number of APCs found on the network branches or in the nodes (representative images, magnification ×10). (E) (i) Overexpression of miR-532-5p produced a significant increase in branch formation compared to control, but no change in the number of cells on branches or nodes was observed (ii), Representative images, magnification ×10; n = 8 biological replicates, *p < 0.05 and **p < 0.01 versus HUVECs, ^+^p < 0.05 versus scrambled-APCs. (F) (i) Inhibition of miR-532-5p decreased HUVEC monolayer resistance, while (ii) miR-mimic increased resistance; n = 5–8 biological replicates. *p < 0.05 versus scrambled. Values are means ± SE.

Having demonstrated ischemia is a strong activator of miR-532-5p expression, we performed *in vitro* assays to determine the effects of miR-532-5p titration on APC-related functions. Silencing and overexpression were achieved by transfecting human APCs with miRVana anti-miR inhibitor and miR mimic, respectively. Anti-miR and miR-mimic negative controls were used to exclude any unspecific effect of transfection on observed functional responses. Effective inhibition and overexpression of intra-cellular miR-532-5p were confirmed by qPCR (Figures 4Ai and 4Aii). We could not find miR-532-5p in APC conditioned media except when the cells were transfected with the miR-mimic (Figure 4Aiii).

We next explored if the titration of miR-532-5p causes autocrine effects in human APCs. Inhibition of miR-532-5p had no effect on APC viability, metabolic activity, proliferation, apoptosis, or migration (Figure 4B), whereas overexpression decreased both metabolic activity and apoptosis (p < 0.05 versus scrambled, for both comparisons) but had no effect on viability, proliferation, or migration (Figure 4C).

We next investigated if forced changes in miR-532-5p expression alter the interaction between APCs and ECs. Interestingly, miR-532-5p inhibition abrogated the APC-induced stimulation of human umbilical vein endothelial cells (HUVECs) network formation on Matrigel, as assessed by measuring the cumulative branch length (p < 0.01 versus scrambled; Figures 4Di and 4Dii). The opposite effect was seen in response to miR-532-5p overexpression, which resulted in potentiation of the stimulatory effect of APCs on network formation (p < 0.01 versus HUVECs alone and p < 0.05 versus HUVECs + Scrambled-transfected APCs; Figures 4Ei and 4Eii). However, no change was observed in the number of APCs sitting on branches or nodes (data not shown). Furthermore, miR-532-5p inhibition decreased HUVEC monolayer resistance (p < 0.05 versus scrambled; Figure 4Fi), while overexpression of miR-532-5p increased resistance (p < 0.05 versus scrambled; Figure 4Fii).

### mir-532-5p Modulates Ang-1 Expression in Human APCs

**Figure 5:**
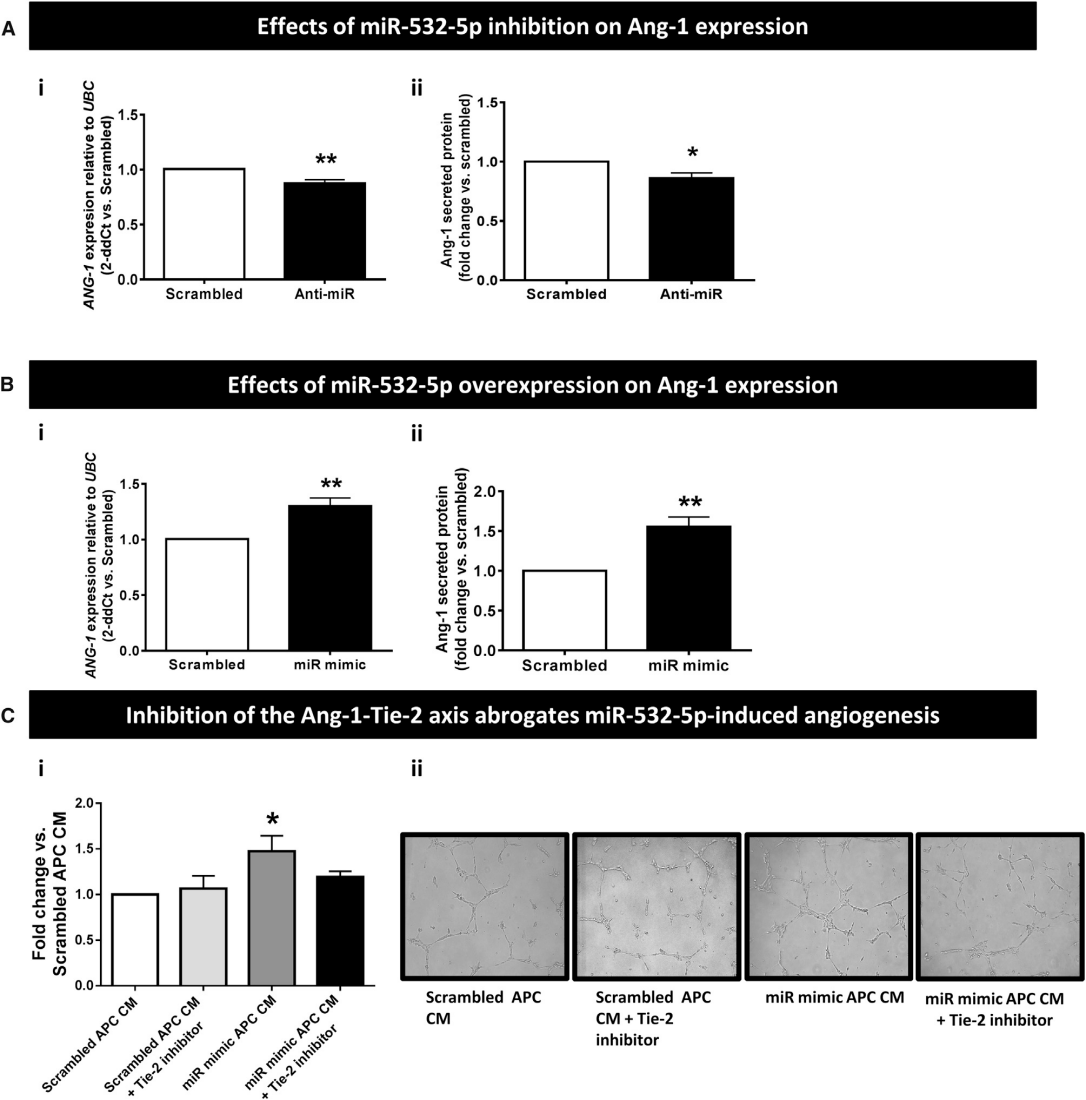
The Ang-1-Tie-2 Axis Is Involved in the Proangiogenic Effect of miR-532-5p (A) Effects of miR-532-5p inhibition on *ANG-1* mRNA (i) and protein levels (ii) in human APCs. (B) Effects of miR-532-5p forced expression on ANG-1 mRNA (i) and protein levels (ii); n = 6 biological replicates per group. *p < 0.05 and **p < 0.01 versus Scrambled. (C) (i) Bar graph showing the effect of Tie-2 inhibition on the inductive effect of miR-532-5p-overexpressing APC conditioned media (CM) on HUVECs network formation. (ii) Representative Matrigel images, ×10 magnification; n = 3 biological replicates per group. *p < 0.01 versus Scrambled-transfected APC CM. Values are means ± SE.

Considering miR-532-5p influences the control of network formation by APCs, we explored the possible participation of Ang-1 and vascular endothelial growth factor A (VEGF-A), which are key angiogenic factors produced by PCs,^2^ in these phenomena. Results indicate the Ang-1/Tie-2 axis is indeed involved in the miR-532-5p-induced promotion of *in vitro* angiogenesis. This is supported by several pieces of evidence. First, miR-532-5p inhibition reduced and miR-532-5p overexpression increased *ANG-1* mRNA and Ang-1 protein levels in human APCs (p < 0.05 to p < 0.01 versus scrambled; Figures 5A and 5B), whereas no changes were seen in *VEGF-A* expression (Figure S2). Second, a Tie-2 inhibitor was able to block the promotion of HUVECs network formation by the conditioned media of miR-532-5p-overexpressing APCs (Figure 5C). On the other hand, inhibition of Tie-2 in miR-532-5p-overexpressing APCs had no effect on apoptosis when measured by caspase assay (data not shown), thus discounting an effect of Ang-1 on the promotion of APC viability by miR-532-5p.

### miR-532-5p Regulates Ang-1 via Inhibition of the Transcription Factor BACH1

miRTarBase predicted *RUNX3*, *TRAPPC2P1*, *METTL20*, *ZFHX3*, and *CPNE1* to be direct targets of miR-532-5p. However, we did not find any change in the expression of the above genes when miR-532-5p expression was inhibited (Figure S3). Western blots to examine protein expression for each gene of interest also showed no regulation (data not shown).

**Figure 6:**
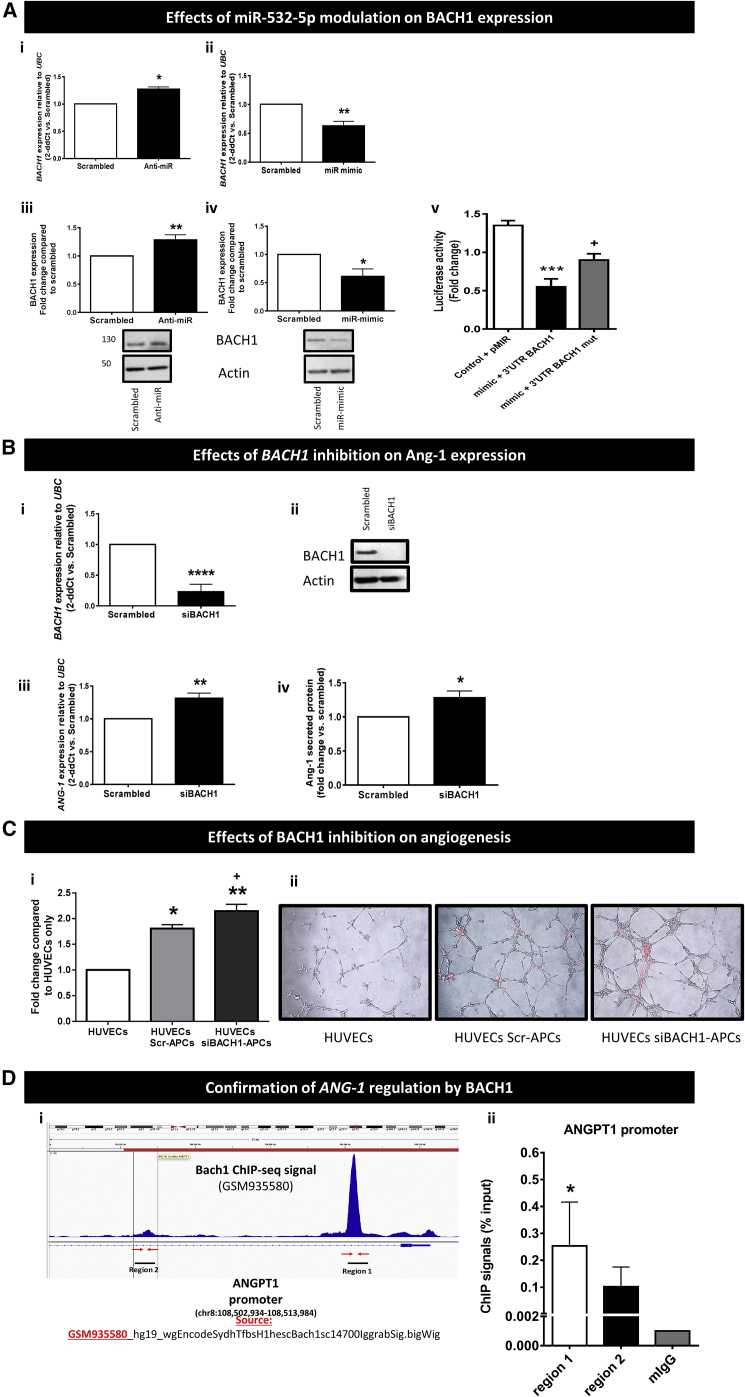
miR-532-5p Regulates *ANG-1* via Inhibition of the Transcription Factor BACH1 (A) qPCR (i and ii) and western blotting (iii and iv) demonstrating the effect of miR-532-5p inhibition (i and iii) (n = 4 biological replicates) and overexpression (ii and iv) (n = 5 biological replicates) on *BACH1* gene and protein expression in APCs. *p < 0.05 and **p < 0.01 versus scrambled. v, Luciferase activity at 48 hr post-co-transfection of HEK293T cells with miR-532-5p mimics and the following plasmids: pMIR reported (empty vector), 3′ UTR-*BACH1*, and 3′ UTR-*BACH1*mut (with mutation of the putative miRNA target site) (n = 5 replicates). ***p < 0.001 versus control, ^+^p < 0.05 versus mimic 3′ UTR *BACH1*. (B) qPCR, n = 4 (i), and representative western blot image, n = 3 (ii), demonstrating siRNA silenced both *BACH1* gene (****p < 0.0001 versus control) and protein expression, respectively. Silencing *BACH1* resulted in an increase *ANG-1* mRNA (n = 6) (iii) and Ang-1 protein (n = 5) (iv). *p < 0.05 and **p < 0.01 versus scrambled. (C) Inhibition of *BACH1* expression produced a significant increase in network formation compared with control (i); representative images, red color: DiL staining of pericytes, magnification ×10. n = 7 biological replicates (ii), *p < 0.05 and **p < 0.01 versus HUVECs, ^+^p < 0.05 versus HUVECs + Scr-APCs. All values are means ± SE. (D) (i) BACH1 transcription factor binding sites by ENCODE ChIP-seq from NCBI GEO: GSM935580 were consulted and subsequent ChIP-qPCR performed for BACH1 occupancy of the ANGPT1 promoter regions (1) chr8:108,507,212–108,509,973 and (2) chr8:108,499,983–108,505,507, transcribed in [−] direction. (ii) ChIP-qPCR analysis confirms the regulation of *ANG-1* by BACH1. qPCR values are expressed as mean ± SE and are representative of n = 3 independently performed qPCR experiments with one biological replicate of APC. *p < 0.05 versus mIgG.

As miR-532-5p is not predicted to target *ANG-1* directly, we performed an *in silico* analysis for gene targets of miR-532-5p that could regulate *ANG-1*. This resulted in three potential candidates, *BACH1*, *HIF1AN*, and *EGLN1*. Modulation of miR-532 by anti-miR or mimic did not affect the expression of either *HIF1AN* or *EGLN1* (Figure S4). In contrast, several lines of evidence identify the transcription factor BACH1 as the key link between miR-532-5p and the Ang-1/Tie-2 signaling pathway. BACH1 (also known as BACH1, basic leucine zipper transcription factor 1) reportedly acts as a hypoxia-inducible repressor of gene expression and regulates important biological processes including oxidative stress, inflammation, apoptosis, cell proliferation, fibrosis, and angiogenesis.^35^ Importantly, qPCR analysis demonstrated that when miR-532-5p was inhibited in human APCs, *BACH1* expression increased (Figure 6Ai). In contrast, when miR-532-5p was overexpressed *BACH1* expression decreased (Figure 6Aii). Concomitant regulation of BACH1 protein expression was confirmed by western blotting (Figures 6Aiii and 6Aiv). The TargetScan 6.2 algorithm predicted *BACH1* contains a single conserved binding sequence for miR-532-5p in its 3′ UTR.^36^ To confirm that miR-532-5p directly binds the 3′ UTR of *BACH1*, we performed a reporter assay in which the luciferase reporter gene was fused to the wild-type 3′ UTR of *BACH1*. Overexpression of miR-532-5p decreased luciferase activity for the putative target gene, whereas mutation of the putative miR-532-5p binding site within *BACH1* prevented the miR-532-5p-induced reduction in luciferase activity (Figure 6Av). Having demonstrated that *BACH1* is under the direct inhibitory control of miR-532-5p, we next asked the question of whether *BACH1* modulation could affect the expression of *ANG-1* in human APCs. Small interfering RNA (siRNA) was used to silence *BACH1* expression, which was confirmed by qPCR and western blot (Figure 6Bi and 6Bii). This resulted in an increase in *ANG-1* RNA levels in APCs (Figure 6Biii) and Ang-1 protein levels in the APC-conditioned media (Figure 6Biv). Furthermore, we investigated the effect of *BACH1* silencing on APC network formation when in co-culture with HUVECs (Figure 6C). BACH1 silencing resulted in an increase of cumulative branch length. To assess if *ANG-1* is a direct target of BACH1-mediated transcriptional regulation, chromatin immunoprecipitation (ChIP) assay coupled with qPCR was performed. (Figure 6D). Utilizing the ENCODE database,^37^ the BACH1 reads distribution from the ChIP-seq NCBI GEO: GSM935580 file was visualized across the human genome in IGV Browser (IGV_2.4.4).^38^, ^39^ The BACH1 reads were assessed at the promoter region of ANGPT1. We visualized one significant peak in chr8:108,507,212–108,509,973 corresponding to active promoter *region1* on *ANG-1*, and a smaller peak in the *region2* upstream (chr8:108,499,983–108,505,507) (Figure 6Di). Genomic sequences for both above binding regions were retrieved from UCSC, and primers were designed. qPCR was performed with the precipitated DNA and validated primers. Binding was detected in both regions with much stronger ChIP-qPCR signal observed in region 1 (Figure 6Dii).

### miR-532-5p Function *In Vivo*

**Figure 7:**
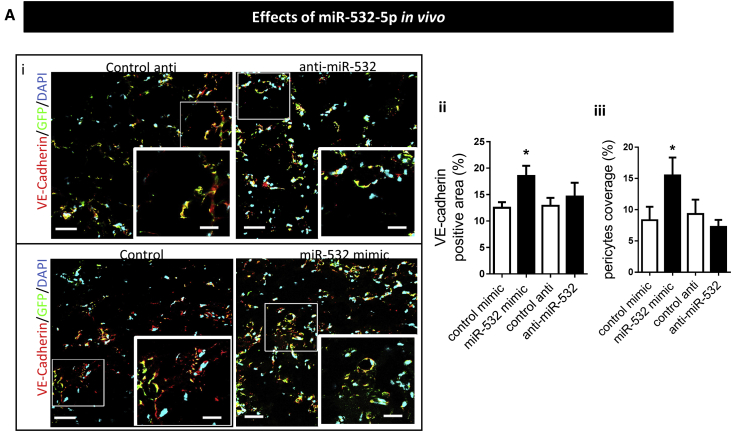
*In Vivo* Matrigel Plug Assay Shows miR-532-p Expression Modifies the APC Ability to Stimulate Angiogenesis *In Vivo* Control, miRNA mimic, or anti-miRNA treated pericytes (GFP-positive) plus HUVECs embedded into Matrigel were implanted subcutaneously into nude mice. (i) Confocal analysis of representative cryostat sections of the Matrigel plug: PCs are stained with an anti-GFP antibody (green) and HUVECs with VE-cadherin antibody (red). Scale bars, 50 μm for large images and 25 μm for the inset; (5 × 5 tiled image). (ii) Results of quantification of areas of endothelium VE-cadherin positive (in percentage in one microscopic view); (iii) ratio of the pericyte-covered endothelium (in percentage). Bars in the graphs represent mean values ± SE, (n = 5) *p < 0.05.

Finally, we determined if modulation of miR-532-5p expression affects the ability of human APCs to participate in the formation of vascular networks upon transplantation *in vivo*. Results show an increase in vessel coverage by miR-532-5p-overexpressing APCs as compared with control APCs, thus suggesting that miR promotes microvascular maturation (Figure 7).

## Discussion

There is a substantial paucity of data regarding the expression and pathophysiological role of miRs in human PCs. We previously showed that miR-132 plays a role in the pro-angiogenic and anti-fibrotic activity of APCs in a murine model of myocardial infarction, respectively, through inhibition of Ras-GTPase-activating protein and methyl-CpG-binding protein 2.^16^ The hypoxia-miR Let-7d was found upregulated in PC-derived neurospheres and proposed to be involved in PC differentiation.^17^ In another study, Caporali et al.^15^ reported the PC uptake of EC-produced miR-503, which, by reducing the expression of EFNB2 and VEGF-A, resulted in an impaired PC migration and proliferation. The present study is the first to investigate the global hypoxia-induced miR profile of human PCs and to identify a number of miRs that are differentially regulated by hypoxia. Nineteen miRs were found to be differentially expressed in response to hypoxia when analyzed using a paired t test. Benjamini-Hochberg correction was also applied to the array data; however, none of the miRs passed this test. This is not surprising, as the sample number tested was small (n = 4), and we observed variation in the amount of miR expression in the samples, though the data trends were similar. The amount of variation between samples when using human samples is a common problem. We performed additional qPCR replicates on a further six patient cell lines to validate the expression of four miRs highlighted by the miRNA array (miR-532-5p discussed above and miR-210, -299-3p, and -339-5p, data not shown). All qPCR results agreed with the initial array data, so we are convinced the results gained from this array are robust. The first four top hits in the list (miR-210, miR-98-5p, miR-192-5p, and let-7f-5p) have been previously associated with angiogenesis^25^, ^26^, ^27^, ^28^ but not directly linked to PCs. Here, we focused on miR-532-5p, the fifth top hit, because this miR was not previously associated with PCs, hypoxia, or angiogenesis.

MiR-532-5p is an intronic miR located in chromosome X and prevalently expressed in brain, cerebellum, and testis. There is a scarce information on miR-532-5p functions and target genes, with seminal studies showing modulation of miR-532-5p in diseases such as cancer,^40^ rheumatoid arthritis,^19^ and deep vein thrombosis.^21^ Another report indicates porcine adipose tissue-derived mesenchymal stem cells secrete vesicle-packaged miR-532-5p.^41^ Furthermore, Bayoumi et al.^22^ recently reported that coronary ECs lacking miR-532-5p exhibit increased transition to a fibroblast-like phenotype via endothelial-to-mesenchymal transition, while ECs overexpressing miR-532-5p display decreased endothelial-to-mesenchymal transition.^22^ Mechanistically, this effect was in part attributed to direct repression of a positive regulator of maladaptive endothelial-to-mesenchymal transition, prss23 (a protease serine 23).

The present study indicates miR-532-5p is regulated by hypoxia and ischemia, though the two conditions exert opposite effects, with the former inducing a modest but significant downregulation, while the latter causing a substantial increase in miR-532-5p. Hypoxia is just one of the many factors that characterize the microenvironment of an ischemic tissue. We tried to reproduce the ischemic condition *in vitro* by exposing APCs to growth factors and chemokines, such as VEGF-A, SDF-1, and interleukin-8 (IL-8), and stressors, such as H_2_O_2_, without succeeding in inducing miR-532-5p expression (P.M., unpublished data). Furthermore, we investigated miR-532-5p expression in response to hypoxia over a range of time points (24, 48, 72, and 96 hr) to see if the miR-532-5p expression was induced. However, no change was seen at 24 hr, while a significant reduction in expression was confirmed at 24 hr and still observed after 72 and 96 hr of hypoxia (P.M., unpublished data). A wider range of time points at the early stages of hypoxia may be more informative. In the *in vivo* limb ischemia model, we observed miR-532-5p expression by skeletal MPCs to be initially increased at the peak of hypoperfusion and then reduced during reperfusion, which may suggest an influence of hemodynamic factors. This possibility is supported by the observation that diabetes, which delays the reperfusion of ischemic tissues, antagonized the upregulation of miR-532-5p in human and murine PCs. The identification of miR-532-5p inducers requires further investigation.

By using inhibitory or mimicry approaches, we showed miR-532-5p exerts both autocrine and paracrine actions. On one hand, the miR promotes pro-survival effects in PCs, and on the other hand, it supports EC network formation and increases vascular permeability. Silencing of miR-532-5p did not alter the expression of predicted target genes *RUNX3*, *TRAPPC2P1*, *METTL20*, *ZFHX3*, and *CPNE1* or the prototypical angiogenic factor *VEGF-A*. Intriguingly, we discovered that miR-532-5p modulates Ang-1 expression. Substantial evidence indicates that the Ang/Tie system is essential for vessel remodeling and for the recruitment of mural cells and consequent vessel maturation during embryonic development and adult vessel homeostasis.^42^, ^43^ Mechanistic studies suggest the participation of Notch signaling through serine/threonine kinase (AKT)-mediated activation of β-catenin.^44^ Preclinical trials using adenoviral-mediated Ang-1 gene transfer^45^, ^46^ or delivery of COMP-Ang-1, a stable and potent Ang-1 variant, showed therapeutic acceleration of tissue healing through potentiation of angiogenesis and lymphangiogenesis.^47^ Intriguingly, despite the important role of Ang-1 in vascular biology, little is known regarding its expressional regulation in human PCs. A recent study showed that hypoxia induces Ang-1 expression in bovine retinal PCs through transcriptional activation by the Hypoxia-inducible factor-2α (HIF2α, also known as endothelial PAS domain protein 1 [EPAS1]).^48^ Therefore, we attempted to understand whether miR-532-5p could be an Ang-1 inducer. To confirm this possibility, we titrated the expression of miR-532-5p and found that inhibition of miR-532-5p decreases Ang-1 expression in APCs, while overexpression of miR-532-5p had the opposing effect. Furthermore, blocking Ang-1 binding to the Tie-2 receptor on ECs resulted in a decrease in network formation when HUVECs were cultured in conditioned media from APCs overexpressing miR-532-5p. However, Ang-1 is not a direct target for miR-532-5p. Using *in silico* analysis, we identified BACH1 as a target of miR-532-5p that could also potentially modulate Ang-1 expression.

BACH1 is reportedly expressed by ECs and plays an inhibitory activity on angiogenesis, though no link with Ang-1 has been reported. *In vitro*, *BACH1* overexpression suppresses EC proliferation and induces cell-cycle arrest and apoptosis by increasing mitochondrial ROS production.^49^ The best characterized molecular mechanism downstream of BACH1 involves the transcriptional suppression of heme oxygenase-1 (HO-1), a hypoxia-induced protein.^50^ In wild-type mice with hindlimb ischemia, *BACH1* forced expression impairs reparative angiogenesis through inhibition of the Wnt/β-catenin signaling pathway. In contrast, *BACH1* knockout mice show an enhanced angiogenic response to ischemia, which translates into improved limb muscle reperfusion as compared to wild-type controls.^51^

In our study, overexpression and inhibition of miR-532-5p were shown to modulate the expression of BACH1 in APCs. The targeting of *BACH1* by miR-532-5p was confirmed using a luciferase reporter assay to prove the 3′ UTR of *BACH1* contains a binding site miR-532-5p, further verifying the *in silico* results. As far as we are aware, this is the first demonstration of a role for miR-532-5p in modulating *BACH1* expression. To confirm miR-532-5p influences targets downstream of BACH1, we designed a target site blocker oligonucleotide sequence complementary to the miR-532-5p binding site on the promoter region of *BACH1*. However, transfection of this sequence into APCs was found to have toxic effects, even at low dosage, a result we report for completeness of the information (P.M., unpublished data). This side effect is a recognized problem with oligonucleotide sequences which contain a phosphorothioate backbone.^52^

The alternative approach of silencing *BACH1* confirmed that the transcription factor acts as a suppressor of Ang-1 expression in APCs, so that when BACH1 is inhibited, *ANG-1* is transcribed and secreted, resulting in potentiation of *in vitro* EC network formation. BACH1 transcriptional repression of *ANG-1* was further confirmed using ChIP analysis, demonstrating that BACH1 occupies two binding regions on the promoter of *ANG-1*. Importantly, this translated into potentiation of *in vivo* angiogenesis, where subcutaneous implantation of miR-532-5p-overexpressing APCs caused an increase in PC coverage of microvessels and greater expression of VE-cadherin, suggesting a role in microvessel maturation.

### Conclusions

This is the first study to show a regulatory pathway for the expression of Ang-1 in PCs. We show compelling evidence for a novel angiogenic mechanism that involves the induction of Ang-1 through miR-532-5p-mediated inhibition of the transcriptional repressor BACH1, leading to modulation of network formation in models of angiogenesis. miR-532-5p has been implicated in the modulation of epithelial-to-mesenchymal transition. Our data indicate that miR could play a role in the transition of PCs from a quiescent to an activated angiogenic phenotype. These results provide important information in the regulation of a key angiogenic pathway and reveal a previously unknown associative role for miR-532-5p and BACH1 in vascular biology, which may translate into new therapeutic avenues in reparative and tumoral angiogenesis.

## Materials and Methods

### Ethics of Human Studies

Studies using human cells were covered by Research Ethics Committee approvals (06/Q2001/197 and 11/2009) and complied with the principles stated in the Declaration of Helsinki. All the subjects gave informed written consent for the experimental use of donated material. APCs were isolated from saphenous vein leftovers from coronary artery graft bypass surgery. MPCs were isolated from biopsy material obtained from different anatomic districts of the lower extremities from subjects referred for investigations or therapeutic interventions related to leg varicosity or suspected bone-related pathologies that then resulted in negative (controls, n = 8) or from sartorius muscles from patients at the occasion of major amputation for CLI without (CLI, n = 3) or with (T2D + CLI, n = 5) superimposed diabetes. CLI was diagnosed according to TASC 2007 i.e., rest pain and/or ulcer or gangrene, transcutaneous oximetry at the dorsum of the foot <30 mmHg and/or ankle pressure <70 mmHg. The cell isolation protocols have been published previously.^2^, ^31^ Patients characteristics are listed in Table S1.

### Cell Culture

APCs were cultured on fibronectin and gelatin in endothelial cell growth medium-2 (EGM-2) with all growth factors and 2% fetal bovine serum (FBS) (Lonza) as previously described.^2^, ^16^ The antigenic profile for APCs was determined by fluorescence-activated cell sorting (FACS) and immunocytochemistry, as previously described.^4^ In brief, cells were fixed with freshly prepared 4% paraformaldehyde (PFA) and probed with the following antibodies: PDGFR-β, VE-cadherin (1:50, Santa Cruz), GATA-4 (1:100), and vimentin (1:400, Abcam) followed by Alexa Fluor 488 anti-rabbit or anti-mouse secondary antibodies (Invitrogen). Isotype negative controls were performed to ensure the immunofluorescence specificity. Cell nuclei were stained with 300 nM DAPI (Thermo Fisher Scientific). Images were acquired with a fluorescent microscope (Olympus BX40) at 40× magnification and merged using ImageJ software. All experiments were performed at passage 7 (P7). To induce hypoxia, APCs were incubated in 2% oxygen (multi-gas incubator, MC0-19M-PE, Panasonic) for the time points described. Conditioned media, protein, and RNA were then collected for further study. HUVECs (Lonza) were grown in EGM-2 with all growth factors and 2% fetal calf serum (FCS). The phenotypical characterization of MCPs was carried out by immunofluorescence staining for the typical PC markers NG2, CD146, and PDGFR-β, the muscle marker CD56 and the endothelial (CD31) and satellite cell (PAX7) markers. Moreover, MPC identity was assessed by flow cytometry analysis of MPCs using anti-ALP antibody (from BD Biosciences). Fluorescence was analyzed in a FACSCanto flow cytometer using the FACSDiva software (BD Biosciences) setting a non-labeled population as negative control.^31^

### miRNA Array

Four independent APC lines were exposed to normoxic (20%) or hypoxic (2%) O_2_ conditions for 48 hr in serum-free EGM-2. Total RNA was extracted as described below. RNA samples were sent to Exiqon to perform a miRCURY LNA Universal RT miR PCR panel. miRNA array amplification was performed on a Roche Lightcycler 480.

### Cell Transfection

Opti-MEM media (Thermo Fisher Scientific) and Lipofectamine 2000 (Invitrogen) was used to transfect APCs with hsa-miR-532-5p miRVana miRNA mimic (MC11553) (final concentration 1 nM), hsa-miR-532-5p anti-miR miRNA inhibitor (AM11553) (both Applied Biosystems), or on-target plus *BACH1* SMARTpool siRNA (L-007750-00-0005, GE Healthcare) (all final concentration 25 nM). miRVana miRNA mimic negative control #1 (4464058), anti-miR miRNA inhibitor negative control #1 (AM17010) (Applied Biosystems), and Silencer Select Negative Control No. 2 siRNA, (Thermo Fisher Scientific) were used as controls (final concentration 25 nM). Overexpression or silencing was confirmed by qPCR. Conditioned media was collected to quantify miRNA and Ang-1 secretion.

### *In Vitro* Assays

#### Hypoxia and Reoxygenation

APCs were cultured under normoxic or hypoxic conditions for 48 hr in EGM-2 without FCS. A further plate of cells was subjected to hypoxia for 48 hr in EGM-2 without FCS, followed by 48 hr in normoxia with EGM-2 containing FCS to replicate an *in vivo* ischemia-reperfusion injury. RNA and conditioned media (CM) were collected.

#### Functional Assays

Cell apoptosis (caspase 3/7, Promega), metabolism (MTS assay, Promega), viability (viability/cytotoxicity assay, Biotium), and proliferation (EdU, Thermo Fisher Scientific) were performed in triplicate according to manufacturer’s instructions.

#### Scratch Assay

Migration was measured on gelatin and fibronectin-coated multi-well plates and manual scratch distance was measured after 18 and 24 hr, as described previously.^2^ Percentage of gap closure (%GAP) was calculated as following: %GAP = 100 − (100*D^1^/D^0^).

#### Endothelial Resistance

The effects of miR-532-5p modulated APC-conditioned media on HUVEC barrier properties was assessed by real-time measurement of transendothelial resistance using an automated ECIS system (ECIS 1600R, Applied Biophysics, NY, USA) as described previously.^53^, ^54^ In brief, HUVECs were seeded into 8-well arrays (10E+, Applied Biophysics) at a density to allow monolayer confluence. Arrays were attached to array holders and allowed to stabilize for 1 hr. Resistance was then measured from each well, every 15 s for 10 min. Media was then removed and replaced with APC-conditioned media. Resistance measurements were made every 15 s for 1 hr.

#### ELISA

Anti-miR- or mimic-treated APC were incubated following transfection with 1 mL of EGM-2 without FBS or VEGF for 48 hr. Conditioned media was collected and secreted Ang-1 levels were analyzed using the manufacturer’s ELISA protocol (Bio-Techne).

#### *In Vitro* Angiogenesis

Promotion of *in vitro* angiogenesis by APCs was assessed using a Matrigel assay (BD Biosciences). HUVECs (Lonza) and APCs were cultured together in a 96-well plate on 70 μL Matrigel in a ratio of 3:1 HUVEC:APC for 6 hr. Total tube length was measured, along with the number of APCs found on nodes and branches. To visualize APCs, the cells were stained with long-term cell tracker VyBrant diI (Life Technologies, UK). DiI was diluted 1:1,000 in PBS and incubated with adherent confluent APCs for 5 min at 37°C and then on ice for a further 15 min in the dark. Cells were then washed with PBS, left to recover for 24 hr, then used for experiments.

To determine the role of Ang-1 in HUVEC network formation, HUVECs were cultured on Matrigel with PC-conditioned media plus a Tie-2 inhibitor (Abcam). In brief, HUVECs in 100 μL EGM-2 were seeded onto 70 μL Matrigel along with either 100 μL serum-free-conditioned media from PCs treated with scrambled sequence or miR-532-5p mimic plus/minus 7.5 μM Tie-2 inhibitor. Cells were incubated for 6 hr, then the total tube length measured.

### RNA Extraction and Quantitative Real-Time Analysis

Total RNA was extracted using miRNeasy kit (QIAGEN). RNA concentration and purity were assessed using a NanoDrop 2000 Spectrophotometer (Thermo Fisher Scientific). A miR Reverse Transcription Kit (Applied Biosystems) along with specific miRNA assay probe *hsa-miR-532-5p* (cat no. 001518) were used to determine miRNA expression. miRNA expression was normalized to *U6* (cat no.001973) (Applied Biosystems). For mRNA analysis, RNA was reverse transcribed using a High Capacity RNA-to-cDNA Kit (Applied Biosystems). cDNA amplification was performed on a QuantStudio 6 Flex (Thermo Fisher Scientific) and normalized to *UBC* (Hs00824723_m1). Expression of *ANG-1* (Hs00375822_m1), *BACH1* (Hs00230917_m1), *VEGFA* (hs00900055_m1), *HIF1AN* (hs00215495_m1), *EGLN1* (Hs00254392_m1), RUNX3 (Hs00231709_m1), TRAPPC2P1 (Hs00249201_m1), METTL20 (Hs00697698_m1), ZFHX3 (Hs00199344_m1), and CPNE1 (Hs00537765_m1) (all Applied Biosystems) was measured. The mRNA expression level was determined using the 2^−Δ*ct*^ method. Each reaction was performed in triplicate.

### Luciferase Assay

To investigate whether miR-532-5p directly regulates BACH1 expression, portions of the 3′ UTR of the potential target gene was inserted downstream of a luciferase open reading frame (pLUC). *BACH1* 3′ UTR vector was purchased from SwitchGear Genomics. Vectors in which five nucleotide mutations were inserted in the 3′ UTR sequences (position 2,982–2,988 of BACH1 3′ UTR) complementary to the miR-532 “seed” sequence were prepared using GeneTailor kit (Invitrogen). Primers are as follows: forward, 5′-TTTAAATGTTTTATTttaattaGTAATAAACTAT-3′, and reverse, 5′-AATTTTCATTCTACCCAACAAGTTTC-3′. Luciferase constructs were transfected into HEK293T cells together with pRenilla vector and either miR-532-5p mimic or a scrambled oligonucleotide sequence (control). Cells were cultured for 48 hr and assayed with the Dual-Luciferase Reporter Assay System (Promega).

### Protein Extraction

Cells were lysed in radioimmunoprecipitation assay (RIPA) buffer containing phosphatase and proteinase inhibitors (1:100, both Sigma). After incubation on ice, the whole APC lysates were centrifuged at 12,000 × *g* for 10 min at 4°C. Protein concentration was quantified using a BCA protein assay (Thermo Fisher Scientific).

### Western Blotting

Protein samples were prepared in Laemmli loading buffer, incubated for 10 min at 95°C, resolved on 7.5% SDS-PAGE, and transferred onto polyvinylidene fluoride (PVDF) membranes (Immobilon-P PVDF Transfer Membrane 0.45 m, Millipore). Membranes were blocked using 5% BSA or 5% non-fat dried milk in Tris-buffered saline (TBS) containing 0.05% Tween 20 (Sigma-Aldrich) for 1 hr at 15°C–25°C. Primary antibodies for BACH1 (1:200, clone F9, Santa Cruz) and actin (loading control, 1:5,000, Clone AC15, Sigma-Aldrich), along with anti-mouse immunoglobulin G (IgG) secondary antibody (1:5,000, GE Healthcare, Thermo Fisher Scientific) were utilized. Membrane development was performed by an enhanced chemiluminescence-based detection method (ECL Prime Western Blotting Detection Reagent, GE Healthcare, Thermo Fisher Scientific) using a ChemiDoc MP system (Bio-Rad). Blot densitometry was analyzed by using the ImageJ 5.1 software.

### ChIP Assay

Nuclei were isolated from formaldehyde (1% final)-fixed APCs by lysing in ChIP Lysis Buffer (1% SDS, 10 mM EDTA, 50 mM Tris-HCL [pH 8.1]) supplemented with protease inhibitors. Chromatin was fragmented by sonication (13 cycles, 60”ON/30”OFF) using a Bioruptor UCD-300 ultrasound sonicator (Diagenode), resulting in 300- to 500-bp DNA fragments visualized on 1% agarose gel. Chromatin was diluted in dilution buffer (1% Triton X-100, 2 mM EDTA [pH 8.0], 150 mM NaCL, 20 mM Tris-HCl [pH 8.1] supplemented with protease inhibitors). DNA-cross-linked proteins were immunoprecipitated in 1-mL reaction (1% kept as input) using 5 μg of BACH1 (sc-271211 X, Santa Cruz) or control mouse IgG antibody. The antibody was pulled down with protein G beads (Dynabeads 10003D, Invitrogen) at 4°C overnight. The beads were separated with a magnetic rack and washed sequentially for 3 min with 1 mL of the following buffers: 3× low-salt wash buffer (0.1% SDS, 1% Triton X-100, 2 mM EDTA, 150 mM NaCl, 20 mM Tris-HCl [pH 8.1]), 2× high-salt wash buffer (0.1% SDS, 1% Triton X-100, 2 mM EDTA, 500 mM NaCl, 20 mM Tris-HCl [pH 8.1]), 1× LiCl wash buffer (0.25 M LiCl, 1% nonidet P-40, 1% sodium deoxycholate, 1 mM EDTA, 10 mM Tris-HCl [pH 8.1]) and 2× Tris EDTA (TE) buffer (10 mM TrisHCl [pH 8.0], 1 mM EDTA). A 10% proportion from the final wash was retrieved, and samples prepared for running on western blot (as described above) to assess the immunoprecipitation (IP) for BACH1 size. Antibody-chromatin complexes were eluted in 200 μL of elution buffer (100 mM NaHCO_3_, 1% SDS, 5 mM NaCl) with vigorous shaking overnight at 65°C. Proteins were digested from the eluate by adding 2 μL of proteinase K (10 mg/mL, Sigma) and incubating for further 2 hr at 65°C. Associated DNA was then purified by extraction using Monarch PCR & DNA Cleanup Kit (T1030S, New England Biolabs). Immunoprecipitated DNA and total input were used as a template for real-time qPCR. The ChIP primers for amplification of region 1 and region 2 are as follows: primer region 1, Fw 5′-CCT TTG GGG CCA TAA GAT TT-3′, Rev 5′-CCG GTC ACA ATC TTT CCA CT-3′; primer region 2, Fw 5′-GCA TGA TTT AAG CCC AGC AG-3′, Rev 5′-CTG GAT TCT TTG AGG GAA CG-3′. ChIP was performed in duplicate using 1 × 10^7^ APCs per sample, and qPCR repeated three times in triplicate. The NEUROD1 promoter region (chr5:134,872,623–134,877,626) was used as a negative control for the absence of BACH1 binding to the promoter, and heme oxygenase-1 (HMOX1 promoter region chr22:35,767,428–35,777,046) used as a positive control.

### Animal Experiments

Experiments were performed in accordance with the Animal (Scientific Procedures) Act (UK) 1986 prepared by the Institute of Laboratory Animal Resources and under the auspices of UK Home Office Project (60/4523) and Personal License (70/25771). Results are reported following the guidelines contained in the Animal Research Report of *In Vivo* Experiments (ARRIVE).

#### Induction of Diabetes and Limb Ischemia

Six- to seven-week-old-male CD-1 mice were made diabetic using STZ (Sigma) or left normoglycemic after STZ buffer administration alone. STZ was delivered intraperitoneally (i.p.) for 5 consecutive days (40 mg kg^−1^ in citrate buffer per day). Fourteen days after the first STZ injection, glycemia after fasting and glycosuria were measured, and only those mice with glycemia above 200 mg dL^−1^ and overt glycosuria entered the protocol. The absence of hyperglycemia and glycosuria in buffer-injected non-diabetic mice was also verified.

Three months after the onset of hyperglycemia, unilateral hindlimb ischemia was surgically induced in anesthetized mice (tribromoethanol, 880 mmol/kg i.p., Sigma) using a refined procedure that consists of ligation (with a 7-0 silk suture) in two points and electrocoagulation of the upper part of the left femoral artery but leaving the femoral vein and nerve untouched. The superficial blood flow of the ischemic and contralateral feet was sequentially analyzed (at 30 min, 3, 7, and 15 days) by color laser Doppler (Moor, USA), and the ratio of blood flow between the ischemic foot and the contralateral foot was calculated and used as an index of % blood flow recovery.

For analysis of vascularization, muscular sections were stained as previously described.^55^ In brief, muscles were excised and whole-mount preparation was performed by fixing in 2% PFA and then embedded in optimal cutting temperature (OCT). Sections were incubated overnight at 4°C with Alexa 488-conjugated isolectin B4 (1:100; Life Technologies) to identify ECs. Slides were observed under a fluorescence microscope (Olympus BX40). The number of capillaries per field was counted, and capillary density was expressed as the number of vessels per mm^2^ of muscular sections.

#### Isolation of NG2-Positive PCs from Mouse Limb Muscles

At 0, 3, 7, and 15 days post-ischemia, adductor muscles were rinsed and digested with collagenase II (Worthington) plus DNase I (Sigma) using gentleMACS Dissociator, following the manufacturer’s protocol. Next, PCs were immunomagnetic sorted using NG2 antibodies (Miltenyi Biotech), as reported.^15^ The purity of cell preparations was analyzed by flow cytometry using NG2-PE (eBioscience, 8012-6504-120, 1:50) antibodies. Expression of miR-532-5p by these cells was then determined by qPCR as described above.

#### Matrigel Plug Assay

To determine the effect of miR-532-5p expression on APCs’ ability to colonize endothelial networks, cold Matrigel was mixed with GFP-labeled APCs (infected with lentiviral vector pLKO-Puro-CMV-GFP at 10 MOI) and transfected with anti-miR, mimic, or respective scrambled sequences (as control). Eight-week-old male CD1 nude mice (Crl:CD1-Foxn1nu; Charles River) were injected subcutaneously into both abdominal flanks with 500 μL growth factor-reduced Matrigel (BD Bioscience) supplemented with fibroblast growth factor (FGF)2 (250 ng/mL; R&D Systems), under anesthesia induced by isoflurane inhalation. Twenty-one days later, the animals were sacrificed and the Matrigel plugs were harvested, fixed in 4% paraformaldehyde at 4°C overnight, and embedded in OCT. Implant sections of 4-μm thickness were incubated overnight at 4°C with the specific endothelial marker VE-cadherin (Santa Cruz), while anti-GPF (Invitrogen) antibody was used to visualize PCs. Sections were then incubated with Alexa 488-conjugated goat anti-rat and anti-rabbit IgG secondary antibodies, respectively (Invitrogen). Nuclei were counterstained with DAPI. Mounted sections were imaged with 20×, 40×, or 100× objective by Zeiss LSM780 confocal microscope. Microvessel density was quantified in 30 fields covering the whole cross-section of the Matrigel plug. The VE-cadherin-positive neovessel area covered with PCs inside implants was quantified using ImageJ software version 7.7.2.

### *In Silico* Analysis

Computational prediction of miR-532-5p target genes was performed using published algorithm TargetScan 7.1.^56^ Analysis of regulatory elements on *ANG-1* promoter was performed using GeneHancer software.^57^ Super-enhancer genomic location chr8:6,424,533–6,430,579 and chr8:6,744,218–6,751,460.

### Statistical Analysis

All data were analyzed using GraphPad Prism software (v6). Comparison of multiple groups was performed by analysis of variance (ANOVA) and Bonferroni post-test multiple comparison tests. A two-group analysis was performed by Student’s t test. Values were expressed as means ± SE. p values less than 0.05 were considered significant.

## Author Contributions

Collection and Assembly of Data, S.C. Slater, E.J., A.M., I.R.-A., T.M., R.V., V.V.A., S.C. Satchell, G.S., A.C.; Data Analysis, A.M., T.M.; Data Analysis and Interpretation, S.C. Slater, S.C. Satchell, G.S., A.C., P.M.; Manuscript Writing, S.C. Slater, P.M.; Provision of Study Material, G.S.; Conception and Design, P.M.; Financial Support, P.M.

## Conflicts of Interest

The authors have no conflict of interest.

## Supplemental Information

Document S1. Figures S1–S4 and Table S1

Document S2. Article plus Supplemental Information
