# Supplementary material for: MicroRNA-532-5p Regulates Pericyte Function by Targeting the Transcription Regulator BACH1 and Angiopoietin-1
Source: Mol Ther. 2018 Sep 1;26(12):2823–37. doi: 10.1016/j.ymthe.2018.08.020 (PMC6277430; doi:10.1016/j.ymthe.2018.08.020)
Supplement: Document S1. Figures S1–S4 and Table S1 [file mmc1.pdf]

YMTHE, Volume *26*

Supplemental Information

MicroRNA-532-5p Regulates Pericyte Function by Targeting the Transcription Regulator BACH1 and Angiopoietin-1

Sadie C. Slater, Eva Jover, Andrea Martello, Tijana Mitić, Iker Rodriguez-Arabaolaza, Rosa Vono, Valeria V. Alvino, Simon C. Satchell, Gaia Spinetti, Andrea Caporali, and Paolo Madeddu

Figure S1

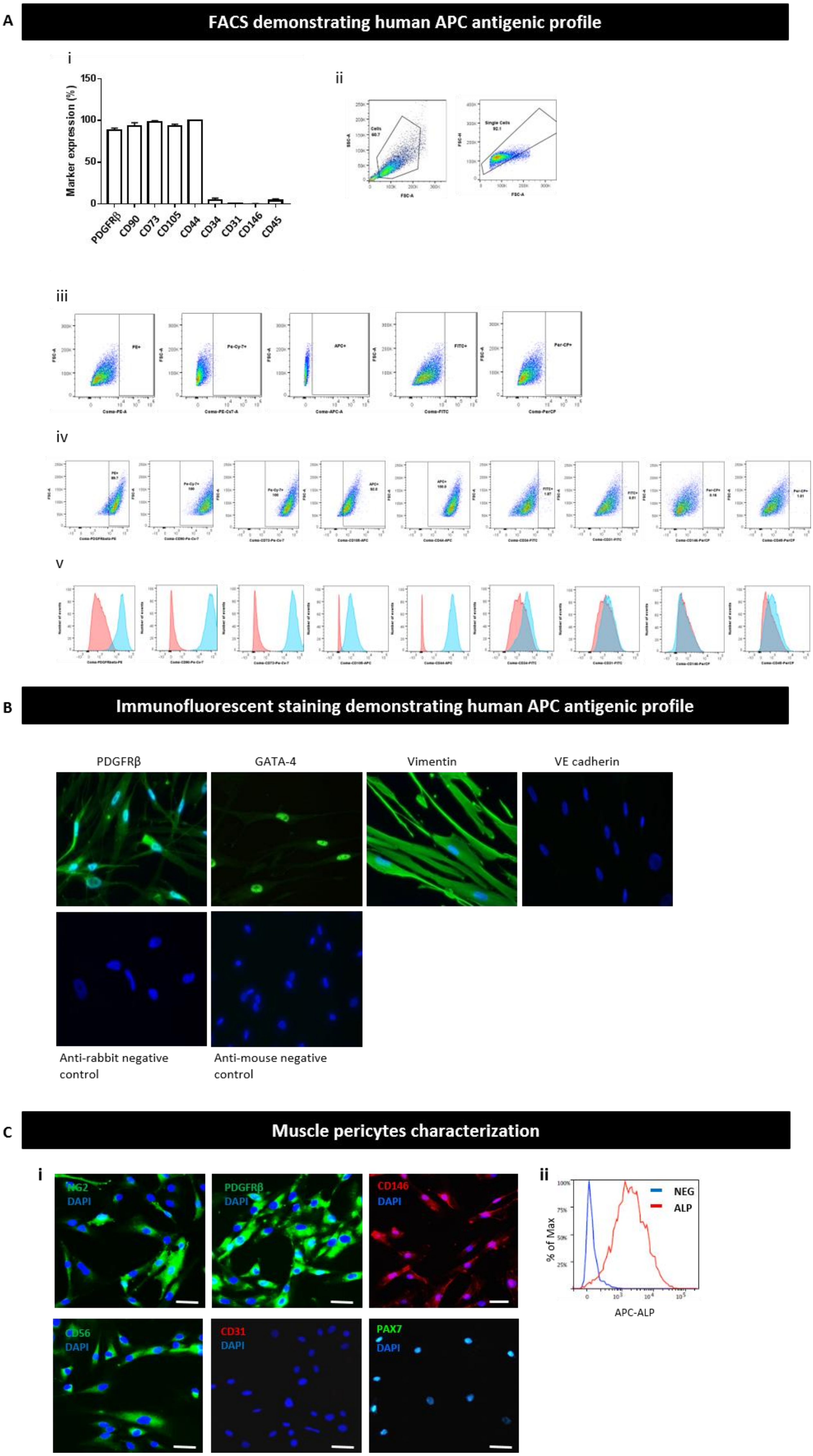

A
FACS demonstrating human APC antigenic profile
i
Marker expression (%)
150
100
50
0
PDGFRβ
CD90
CD73
CD105
CD44
CD34
CD31
CD146
CD45
ii
iii
iv
v
B
Immunofluorescent staining demonstrating human APC antigenic profile
PDGFRβ
GATA-4
Vimentin
VE cadherin
Anti-rabbit negative control
Anti-mouse negative control
C
Muscle pericytes characterization
i
NG2
DAPI
PDGFRβ
DAPI
CD146
DAPI
CD56
DAPI
CD31
DAPI
PAX7
DAPI
ii
% of Max
NEG
ALP
APC-ALP

Supplemental Figure 1. S1A: Flow cytometry analyses of adventitial pericytes isolated from human saphenous veins. (**i**). Bar graphs show the mean±SE values of three human adventitial pericytes (APC) lines. Representative images of flow cytometry gating procedure for a single APC line at P5 are shown. (**ii**) Total cell populations and the single cells (singlets), were gated according to FSC-A vs. SSC-A and FSC-A vs. FSC-H parameters. Cells were further gated for negative (**iii**) and positive controls (**iv**) of selected antigens. Pericyte, mesenchymal, endothelial, and hematopoietic markers were studied. (**v**) Flow cytometry histograms for each surface marker in representative human APC line. Negative control staining profiles are shown by the red histograms, whereas specific antibody staining profiles are shown by light blue histograms. **S1B: Immunofluorescent analyses of APCs isolated from human saphenous veins.** Representative images from a single APC line stained for PDGFRβ, GATA-4, vimentin and VE-cadherin. x40 magnification. Blue staining: DAPI, green staining: protein of interest. **Figure S1C: Characterization of muscular pericytes** Skeletal muscle pericytes (MPCs) were isolated from human sartorius muscle biopsies. The phenotypical characterization was carried out by immunofluoresce staining (**i**) for the typical pericyte markers NG2, CD146 and PDGFRβ. In addition, MPCs show positivity for the muscle marker CD56 and negativity for the expression of the endothelial (CD31) and satellite cell (PAX7) markers. (**ii**) FACS analysis shows that MPCs express alkaline fosfatase (ALP- red line). The blue line shows the negative control (NEG) as a reference.

Figure S2

Effect of miR-532-5p modulation on *VEGF-A* gene expression

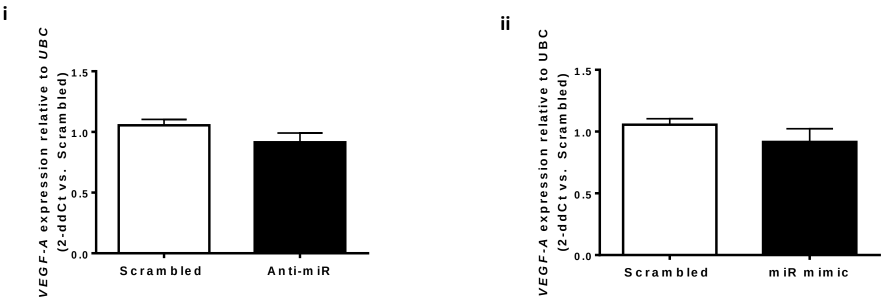

Supplemental Figure 2. Effect of miR-532-5p modulation on VEGF-A expression: (**i**) *VEGFA* mRNA levels in APCs transfected with scrambled sequence or miR-532-5p antimiR. (**ii**) *VEGFA* mRNA levels in APCs transfected with scrambled sequence or miR-532-5p mimic; N=5 biological replicates per group. P=N.S. for both comparisons. All values are means±SE.

Figure S3

qPCR validation of predicted miR-532-5p target genes

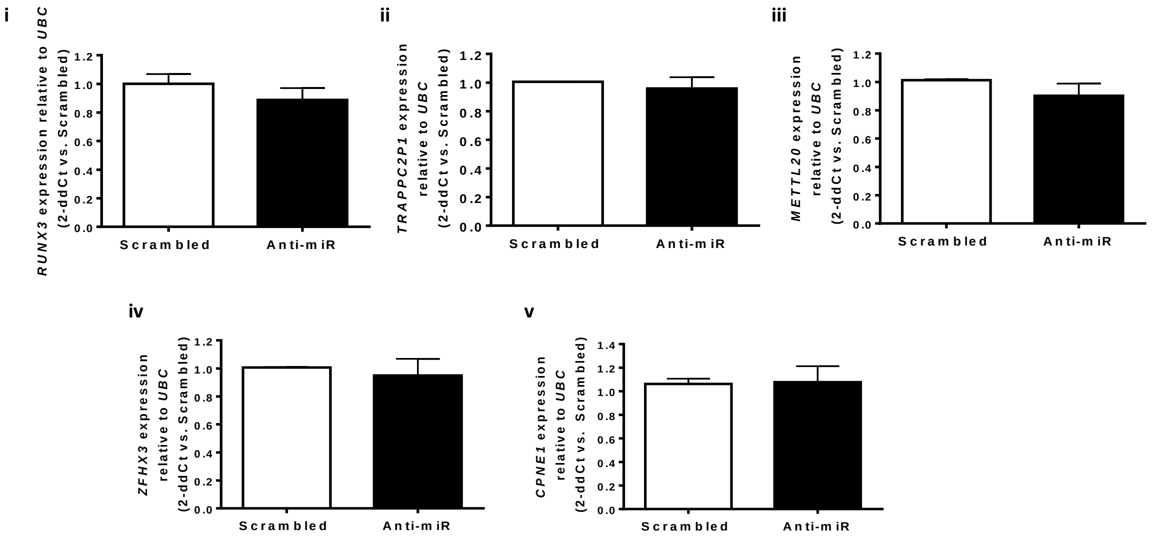

Supplemental Figure 3. Inhibition of miR-532-5p does not affect the expression of genes predicted by in silico analysis: (**i**) *RUNX3*, (**ii**) *TRAPPC2P1*, (**iii**) *METTL20*, (**iv**) *ZFHX3* or (**v**) *CPNE1* expression remain unaltered upon miR-532-5p inhibition. P=N.S. for any of the tested genes; N=6-8 biological replicates. All values are means±SE.

Figure S4

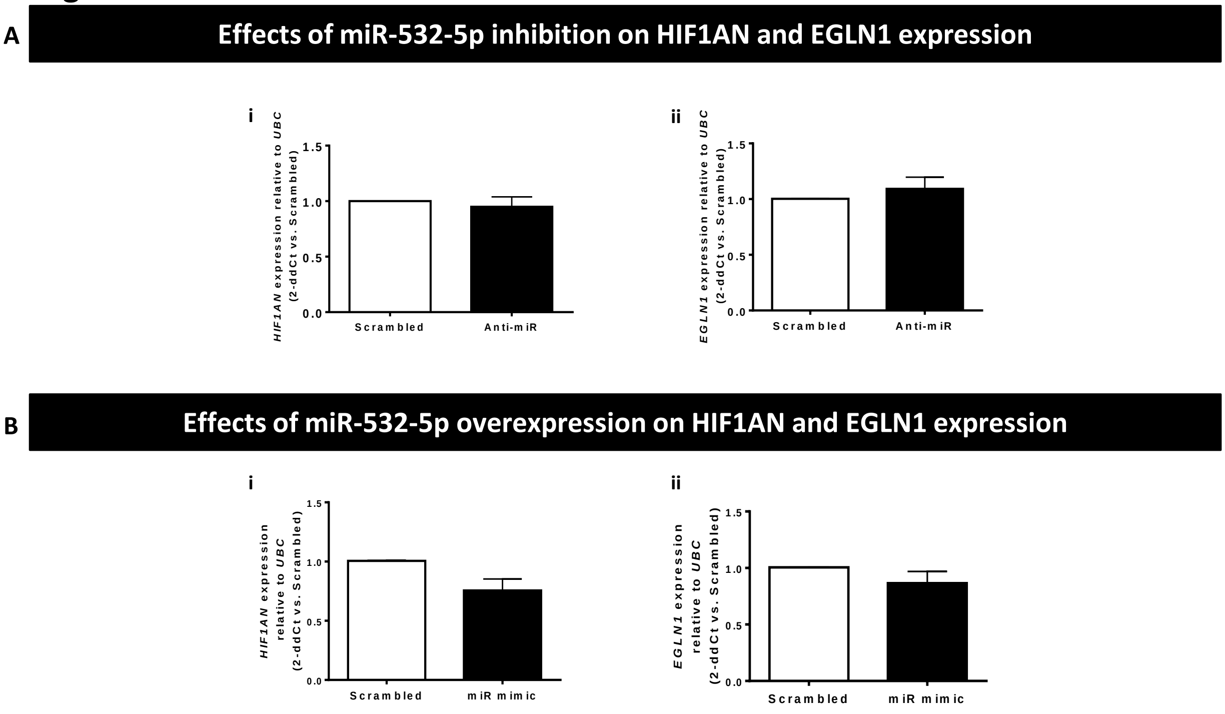

Supplemental Figure 4. Validation of *in silico* predicted targets for the miR-532/Ang-1 axis. *In silico* analysis identified BACH1, HIF1AN and EGLN1 as possible regulators. qPCR demonstrated (**A**) inhibition of miR-532 had no effect on *HIF1AN* (**i**) or *EGLN1* (**ii**) expression. (**B**) Likewise, overexpression of miR-532 had no effect on *HIF1AN* (**i**) or *EGLN1* (**ii**) expression; N=3 replicates. All values are means±SE

Supplemental table 1

Patient ID	Age	Gender	Pathology								
APC			Smoker	DM	MI	HPT	HPC	CPD	CHF	AF	PCI
**5.4.13H**		M									
**15.4.13**		M	Yes	Yes	No	Yes	No	No	No	No	No
**29.5.13**	85	M	Ex >1 month	Yes	Yes	Yes	Yes	Yes	No	Yes	No
**12.6.13**	71	M	Ex >1 month	No	No	Yes	No	No	No	No	No
**19.6.13**		M	Ex >1 month	No	Yes	Yes	Yes	Yes	No	No	No
**9.7.13D**	59	M	Ex >1 month	Yes	Yes	Yes	Yes	No	No	No	No
**15.11.13C**		M	Ex >1 month	No	Yes	Yes	Yes	No	No	No	No
**15.12.13B**		M									
**8.5.14D**	40	M	Yes	No	Yes	Yes	Yes	No	No	No	No
**30.4.14C**	75	M	No	No	No	Yes	Yes	No	No	No	No
**30.4.14G**	63	M	Ex >1 month	No	No	Yes	Yes	No	No	No	No
**8.7.14A**	61	M	Ex >1 month	No	Yes	Yes	Yes	No	No	No	No
**16.10.14A**	67	M	Ex >1 month	No	No	Yes	Yes	No	No	No	No
**28.10.14C**	82	M	No	No	No	Yes	Yes	No	No	No	No
**9.2.15B**	82	M	No	No	Yes	Yes	Yes	No	No	No	No
**20.2.15B**	67	M	Yes	No	Yes	Yes	Yes	Yes	No	No	No
**13.3.15A**	56	M	Yes	No	Yes	Yes	No	No	No	No	No
**30.3.15A**	79	M	Ex >1 month	No	Yes	No	Yes	No	No	No	No
**29.4.15C**	63	M	No	No	Yes	Yes	Yes	No	No	No	Yes
**10.6.15B**	69	M	Ex >1 month	No	Yes	Yes	Yes	No	No	No	No
**15.9.15B**	64	M	Ex >1 month	No	Yes	Yes	Yes	No	No	No	No
**15.9.15C**	41	M	No	No	No	No	Yes	No	No	No	No
**17.11.15B**	67	M	Yes	No	Yes	No	Yes	No	No	No	No
**13.10.15E**	72	M	Ex >1 month	No	Yes	Yes	Yes	No	No	No	No
**12.1.16A**	80	M	Ex >1 month	Yes	No	Yes	Yes	No	No	No	Yes
**9.2.16B**	73	M	Ex >1 month	No	Yes	Yes	Yes	No	No	No	No
**9.2.16C**	57	M	No	Yes	Yes	Yes	Yes	No	No	No	No
**5.3.16A**	64	M	Yes	No	No	Yes	Yes	No	No	No	No
**1.4.16B**	80	M	Yes	No	No	Yes	Yes	No	No	No	No
**5.4.16A**	71	M	No	No	No	Yes	Yes	No	No	No	No
**4.10.16B**	75	M	Ex	No	Yes	Yes	Yes	No	No	No	No
**4.10.16C**	57	M	Yes	No	Yes	Yes	Yes	No	No	No	Yes

Muscle pericytes donors			
**Patient ID**	**Age**	**Gender**	**Pathology**
**MP36**	34	M	CTRL
**MP37**	53	F	CTRL
**MP40**	44	M	CTRL
**MP 20**	58	F	CTRL
**MP 46**	46	M	CTRL
**MP57**	57	F	CTRL
**MP 67**	67	F	CTRL
**MP 73**	73	F	CTRL
**MP 7**	51	M	T2D+CLI
**MP 10**	76	M	T2D+CLI
**MP12**	84	F	T2D+CLI
**MP18**	67	F	T2D+CLI
**MP22**	86	M	T2D+CLI
**MP14**	80	M	CLI
**MP23**	77	M	CLI
**MP24**	85	F	CLI

Table S1. Clinical characteristics of patient donors. APCs and MPCs were isolated as previously described. *Abbreviations*: Ex = Ex-smoker (patient that stopped smoking at least one month before the surgery); DM = Diabetes Mellitus; MI = Myocardial Infarction; HPT = Hypertension; HPC = Hypercholesterolemia; CPD = Chronic Pulmonary Disease; CHF = Congestive Heart Failure; AF = Atrial Fibrillation; PCI = Percutaneous Coronary Intervention; CTRL = no pathology; CLI = critical limb ischemia; T2D+CLI = type 2 diabetes + CLI.
